# *Borrelia miyamotoi* Infection in Patients from Upper Midwestern United States, 2014–2015

**DOI:** 10.3201/eid2208.151878

**Published:** 2016-08

**Authors:** Dean A. Jobe, Steven D. Lovrich, Darby G. Oldenburg, Todd J. Kowalski, Steven M. Callister

**Affiliations:** Gundersen Health System, La Crosse, Wisconsin, USA

**Keywords:** Borrelia miyamotoi, midwestern United States, human infection, bacteria, ticks, vectorborne infections

## Abstract

We confirmed *Borrelia miyamotoi* infection in 7 patients who had contracted an illness while near La Crosse, Wisconsin, USA, an area where *Ixodes scapularis* ticks are endemic. *B. miyamatoi* infection should now be considered among differential diagnoses for patients from the midwestern United States who have signs and symptoms suggestive of tickborne illness.

The upper midwestern United States, which includes the region surrounding La Crosse, Wisconsin, is a well-described focus of *Ixodes scapularis* ticks. As a result, annual incidence of Lyme disease has been high (*1*) and prevalence of human granulocytic anaplasmosis (*2*) and babesiosis has been increasing (*3*). At the Gundersen Health System in La Crosse, we therefore use multiple laboratory procedures to screen patients with suspected *I. scapularis* tick–associated illness; the procedures include a whole-cell *Borrelia burgdorferi* ELISA and Western blot for confirming Lyme disease and PCRs for detecting infection with *Anaplasma phagocytophilum* or *Babesia microti*. However, other human illnesses caused by pathogenic microorganisms transmitted from *I. scapularis* ticks continue to emerge. Primary among them is *Borrelia miyamotoi* (*4*), a spirochete bacterium that colonizes blood and typically causes relapsing fevers accompanied by chills, headache, fatigue, myalgia, arthralgia (*5*–*8*), and (rarely) meningoencephalitis (*9*).

The first case of human infection with *B. miyamotoi* was documented in 2011 in a patient from Russia (*5*); additional cases have since been detected in Russia, Europe, Asia, and the United States (*7*–*11*). Although cases seem to have occurred only in patients who contracted the organism from infected *I. scapularis* ticks in the northeastern United States (*8**,**10**,**11*), persons in the upper midwestern United States (upper Midwest) are also commonly bitten by these ticks. In addition, Hamer et al. (*12*) detected *B. miyamotoi* DNA in *I. scapularis* ticks that had been captured from several sites in the upper Midwest during the 2006 and 2007 tick-questing seasons. We therefore conducted a retrospective investigation to determine whether patients at our healthcare facility with clinically suspected illness caused by tick bite could be infected with *B. miyamotoi*. To do so we also conducted *B. miyamotoi* DNA testing on blood samples submitted for *A. phagocytophilum* or *B. microti* DNA testing.

## The Study

A total of 2,150 DNA samples were obtained from blood of patients evaluated during 2014–2015 for illness and tested for either *A. phagocytophilum* or *B. microti* because of clinical complaints or abnormalities suggestive of tickborne illness. Because our current laboratory procedures for confirming either infection require extraction of DNA from a blood sample before PCR testing, we also evaluated the extracted DNA for *B. miyamotoi*. Ethics approval for detecting and sequencing unique bacterial DNA from routine clinical samples and linkage to patient data without individual informed consent was obtained from the Gundersen Institutional Review Board with the stipulation that patient identifiers be appropriately redacted and information be used only as surveillance data for public health purposes.

We initially screened the DNA samples with a modification of a previously described *B. miyamatoi* screening PCR (*13*), which amplified a 70-bp DNA fragment of the 16S ribosomal RNA gene specific for *B. miyamatoi*. In brief, we combined 5 μL of extracted DNA with 20 μL of a master mix that contained 12.5 μL of buffer (AmpliTaq Gold DNA Polymerase with GeneAmp 10X PCR Gold Buffer; Life Technologies, Austin, TX, USA); 2.5 mmol/L magnesium chloride, deoxynucleoside triphosphates; 7 μL of a primer/probe mix comprising forward primer 5′-GCTGTAAACGATGCACACTTGGT-3′, reverse primer 5′-GGCGGCACACTTAACACGTTAG-3′, and probe 5′-HEX-CGGTACTAACCTTTCGATTA-3′; and 0.5 μL (1.5 U) AmpliTaq Gold DNA Polymerase under the following conditions: 1 cycle at 95°C for 10 min, 45 cycles at 95°C for 15 s, 63°C for 1 min, and a final cycle at 25°C for 5 s. For a positive control, we used strain HS1 (ATCC 35209) of *Borrelia hermsii* because *B. miyamotoi* DNA or viable spirochetes are not easily obtained.

We amplified *B. miyamotoi* DNA from 7 patients (1 male, 6 female) whose previous anaplasmosis and babesiosis test results were negative. Mean patient age was 51 years (range 3–70 years), and 3 patients confirmed that they had been bitten by a tick suspected to be *I. scapularis* within the 10 days before becoming ill. The patients also resided in or near La Crosse; although none reported recent travel outside the region, we cannot rule out the possibility that the infections were acquired elsewhere. Further supporting local acquisition, however, we also recently detected *B. miyamotoi* DNA by the PCR used in this study in a small number (≈2%) of *I. scapularis* ticks collected locally (data not shown).

The clinical signs and symptoms strongly supported PCR positivity for *B. miyamotoi* (*5**–**8*). The most common clinical sign was fever; 1 patient reported additional episodes of fever during the 2 months before seeking treatment (Table). In addition, skin lesions were not detected, although 1 patient may have been co-infected with *B. burgdorferi*; an IgM Western blot test performed in the clinical laboratory also yielded reactivity sufficient to provide serodiagnostic confirmation of early Lyme disease (*14*). Moreover, elevated levels of serum alanine aminotransferase (n = 2) or aspartate aminotransferase (n = 1) were detected in the only 2 patients for whom *B. miyamotoi* DNA was identified and who were tested for these liver enzyme abnormalities. In addition, the infected patients received doxycycline therapy for at least 7 days (mean 10 days; range 7–14 days), which uniformly resulted in complete resolution of clinical signs and symptoms.

**Table:** Clinical manifestations in 7 *Borrelia miyamotoi–*infected patients from the midwestern United States, 2014–2015

**Manifestation**	**No. (%) patients**
**Fever**	6 (86)*
**Chills**	3 (43)
**Myalgia**	3 (43)
**Fatigue**	3 (43)
**Arthralgia**	2 (29)
**Headache**	1 (14)
**Nausea**	1 (14)
**Stiff neck**	1 (14)
**Vomiting**	1 (14)
**Skin lesion**	0

*Fever was recurrent for 1 patient.

For final confirmation of infection with *B. miyamotoi*, we then reamplified a 142-bp fragment (bp 636–777) of the glycerophosphodiester phosphodiesterase (*glpQ*) gene from the remaining DNA sample from each patient positive for *B. miyamotoi* by PCR. The *glpQ* gene was targeted because the GlpQ protein is absent in the *Borrelia* species that cause Lyme disease (*15*), which are detected relatively frequently in patients from the region (*1*). In brief, 5 μL of DNA was combined with 20 μL of a master mix that contained 12.5 μL of buffer, 0.5 μmol/L of forward (5′-GATAATATTCCTGTTATAATGC-3′) and reverse (5′-CACTGAGATTTAGTGATTTAAGTTC-3′) primers, and 0.5 μL (1.5 U) of AmpliTaq Gold DNA polymerase. The DNA was then amplified under the following conditions: 1 cycle at 95°C for 3 min followed by 50 cycles at 95°C for 45 s, 56°C for 30 s, and 72°C for 60 s, followed by a final extension at 72°C for 7 min. Amplified material was purified (QIAquick PCR Purification Kit; QIAGEN, Hilden, Germany) and forwarded to Laragen Inc. (Culver City, CA, USA) for sequencing. In each instance, the sequence of the amplified fragment was 100% homologous with that of *B. miyamotoi* LB-2001 (GenBank accession no. CP006647.2).

## Conclusions

Researchers have documented human illness caused by *B. miyamotoi* transmitted from *I. scapularis* ticks (*5*,*7*–*9*,*11*), but infections in patients from the United States have to date been described only for residents of the northeastern part of the country (*10*). In this study, however, we confirmed characteristic illness caused by infection with *B. miyamotoi* in 7 patients who resided in the *I. scapularis* tick–endemic focus surrounding La Crosse, Wisconsin. Given these findings, clinicians in the upper Midwest must now also consider the possibility of *B. miyamotoi* infection in patients with suspected tickborne illness, especially because even more cases will probably be detected as appropriate methods, such *B. miyamotoi*-specific anti-GlpQ antibody testing (*8*,*15*) or *B. miyamotoi* DNA detection, become more widely available. Studies to provide additional insight into human infection with *B. miyamotoi* remain necessary because the prevalence of the illness will probably increase even more as *I. scapularis* ticks continue to disperse.
